# Supplementary material for: A 20-feature radiomic signature of triple-negative breast cancer identifies patients at high risk of death
Source: NPJ Breast Cancer. 2025 Jul 26;11:79. doi: 10.1038/s41523-025-00790-3 (PMC12297616; doi:10.1038/s41523-025-00790-3)
Supplement: Supplementary file 1 — _revised_27.5.25 [file 41523_2025_790_MOESM1_ESM.pdf]

Supplementary File 1

Table S1: Transcriptomic 50-gene signature coefficient values

ENSG	Gene Name	coefficients
**ENSG00000198216**	CACNA1E	-1.983463948
**ENSG00000138684**	IL21	-1.136916621
**ENSG00000112139**	MDGA1	-1.118989171
**ENSG00000204531**	POU5F1	-1.002805636
**ENSG00000188612**	SUMO2	-0.877023874
**ENSG00000171612**	SLC25A33	-0.795862628
**ENSG00000131069**	ACSS2	-0.740184315
**ENSG00000101222**	SPEF1	-0.636621006
**ENSG00000197992**	CLEC9A	-0.620721488
**ENSG00000148396**	SEC16A	0.549946587
**ENSG00000163412**	EIF4E3	-0.54545458
**ENSG00000120437**	ACAT2	-0.486097853
**ENSG00000163132**	MSX1	0.481195052
**ENSG00000198944**	SOWAHA	-0.417367947
**ENSG00000112877**	CEP72	-0.376720224
**ENSG00000102317**	RBM3	-0.310508642
**ENSG00000113231**	PDE8B	0.300226148
**ENSG00000164050**	PLXNB1	-0.298596617
**ENSG00000090534**	THPO	0.284468191
**ENSG00000168246**	UBTD2	-0.213617982
**ENSG00000164879**	CA3	0.210114721
**ENSG00000175354**	PTPN2	-0.192592563
**ENSG00000126233**	SLURP1	0.192260011
**ENSG00000174332**	GLIS1	0.187297665
**ENSG00000164309**	CMYA5	-0.182407307
**ENSG00000073756**	PTGS2	-0.163598889
**ENSG00000163491**	NEK10	-0.161733706
**ENSG00000211445**	GPX3	0.156034835
**ENSG00000153832**	FBXO36	-0.155817545
**ENSG00000075461**	CACNG4	-0.154525625
**ENSG00000257365**	FNTB	0.132524893
**ENSG00000058091**	CDK14	-0.125235734

**ENSG00000130487**	KLHDC7B	-0.121155713
**ENSG00000115041**	KCNIP3	0.11140441
**ENSG00000173947**	PIFO	-0.068849388
**ENSG00000177494**	ZBED2	-0.045333444
**ENSG00000170577**	SIX2	0.034907247
**ENSG00000140379**	BCL2A1	-0.004337739
**ENSG00000164778**	EN2	0.876036128
**ENSG00000158748**	HTR6	0.593968455
**ENSG00000047056**	WDR37	-0.522756608
**ENSG00000158485**	CD1B	-0.374454323
**ENSG00000092020**	PPP2R3C	-0.287403606
**ENSG00000093072**	ADA2	-0.251209125
**ENSG00000205846**	CLEC6A	-0.233715053
**ENSG00000162139**	NEU3	-0.227635217
**ENSG00000145632**	PLK2	0.071480094
**ENSG00000132467**	UTP3	-0.049545935
**ENSG00000150681**	RGS18	-0.039549536
**ENSG00000108384**	RAD51C	0.031872796

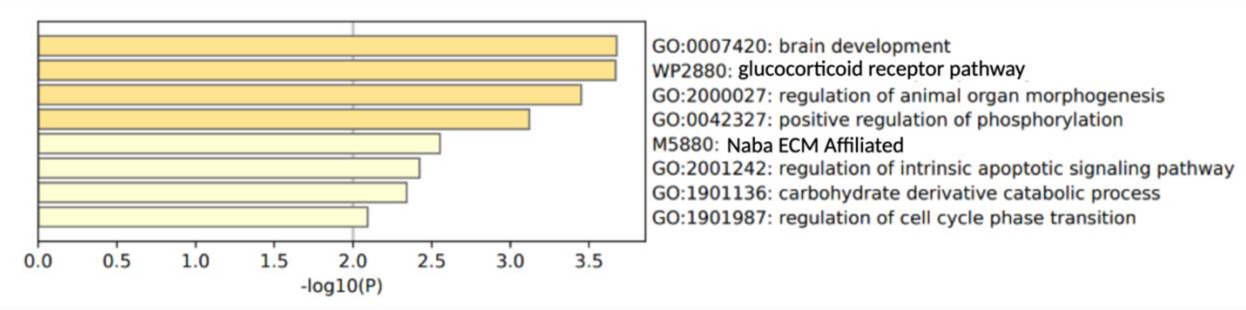

Figure S1. Pathway enrichment analysis of 50 genes in the Transcriptomic Signature set. Bar plot representing the top enriched pathway terms. The x-axis shows the -$\log_{10}$(p-value), indicating statistical significance of enrichment.

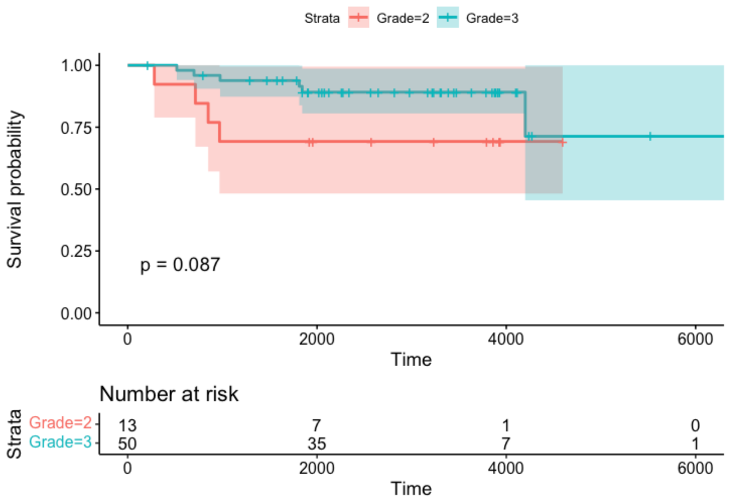

Figure S2. Prognostic effects of tumor grade in the Institutional-Transcriptomic cohort (n=63). Kaplan–Meier survival curves stratified by grade 2 vs grade 3. Log-rank test was used for analysis and a *p*-value < 0.05 for statistical significance

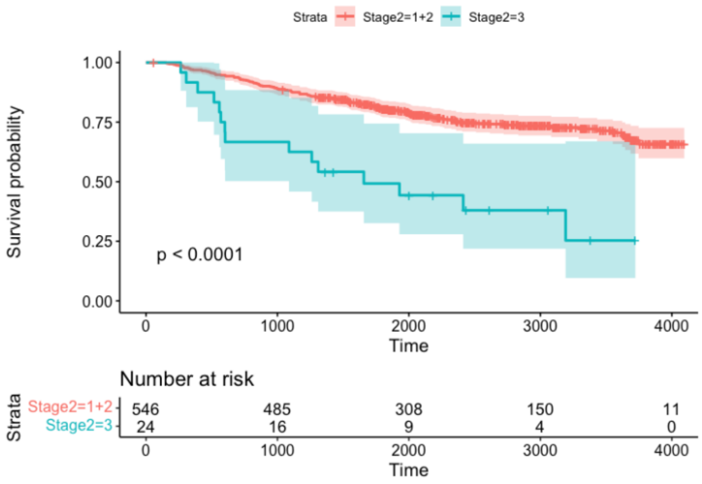

Figure S3. Prognostic effects of tumor stage in the SCAN-B cohort. Kaplan–Meier survival curves stratified by stage I/II vs stage III. Log-rank test was used for analysis and a *p*-value < 0.05 for statistical significance

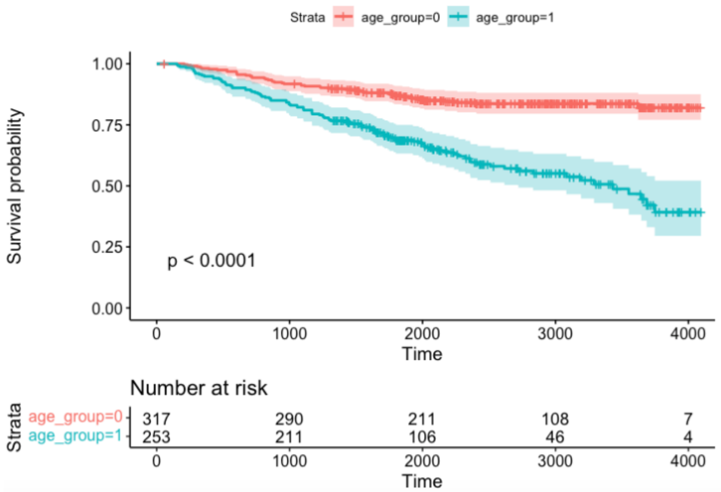

Figure S4. Prognostic effects of patient age in the SCAN-B cohort. Kaplan–Meier survival curves stratified by median age of 65. Log-rank test was used for analysis and a *p*-value < 0.05 for statistical significance

Table S2 Comparison of clinical features between TS-High and TS-Low risk groups in the SCAN-B cohort

	Prognostic Risk Group		
**Variable**	**TS-High (n=265)**	**TS-Low (n=268)**	**p-value**
**Age at diagnosis, mean ± SD (years)**	63.20 ± 15.74	62.95 ± 15.32	0.844 †
**Grade, n (%)**			
Grade 1+2	45 (17.0%)	41 (15.3%)	0.681 ‡
Grade 3	220 (83.0%)	227 (84.7%)	
**Stage, n (%)**			
Stage I+II	253 (95.5%)	257 (95.9%)	0.978 ‡
Stage III	12 (4.5%)	11 (4.1%)	

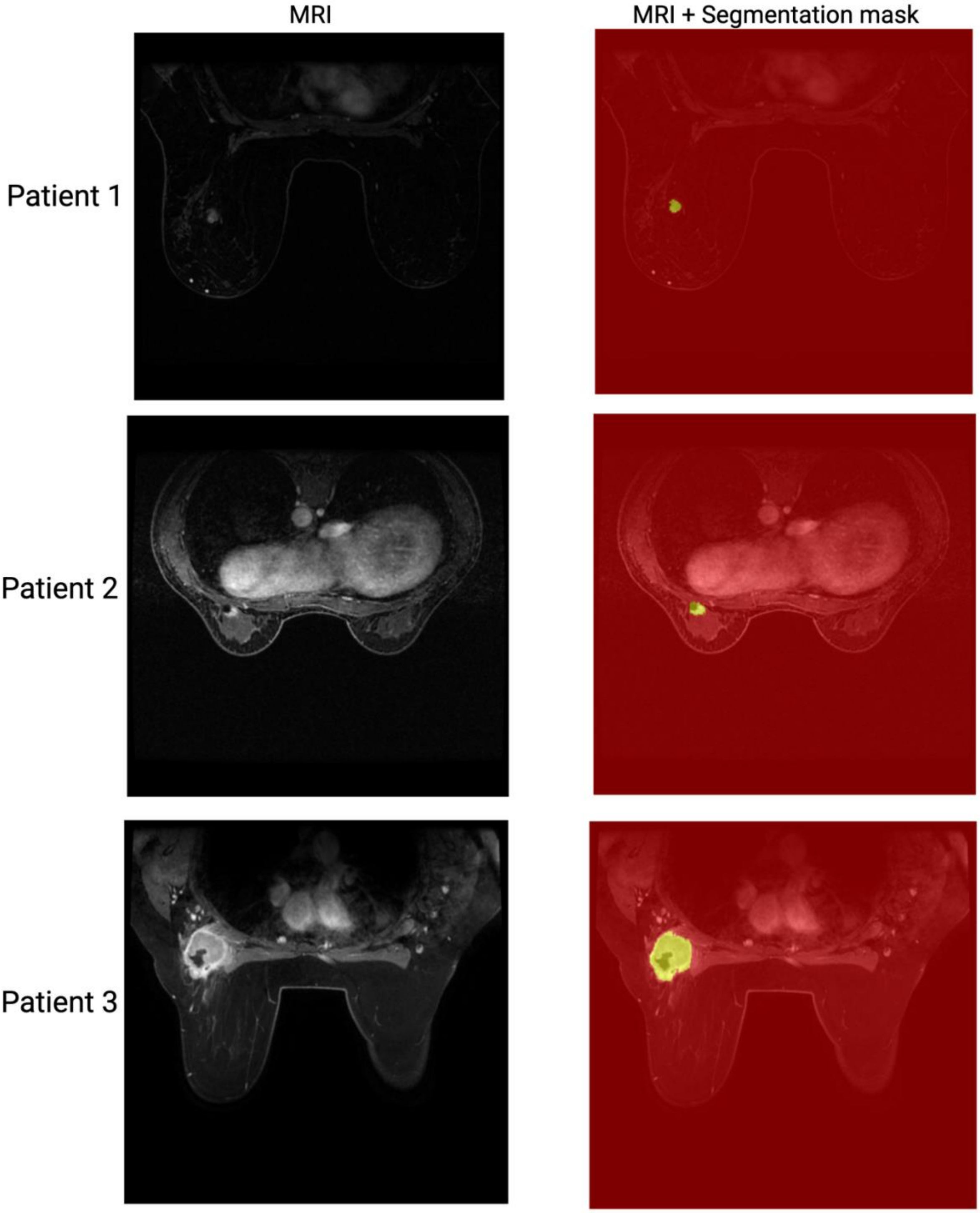

Figure S5. Representative examples of tumor segmentation in breast MRI scans from three patients. The left column displays the original axial MRI images for Patients 1, 2, and 3. The right column shows the corresponding MRI images overlaid with the automated segmentation masks. Tumor regions identified by the segmentation algorithm are highlighted in yellow, overlaid on a red background mask to enhance visibility.

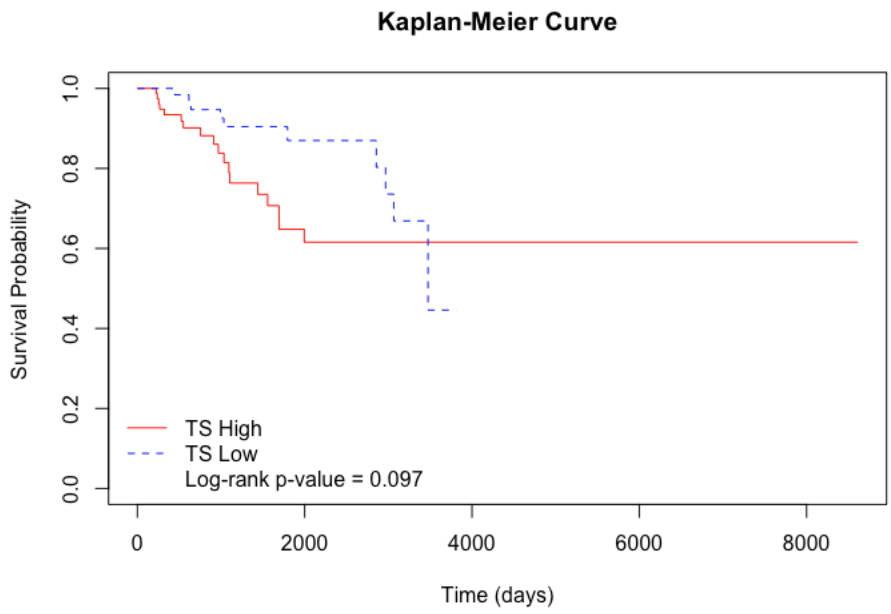

Fig S6. Kaplan-Meier survival curves stratified by TS-high versus TS-low risk groups in the TNBC subset of the TCGA-Breast Cohort (n=172).
